# Trends of domestic violence against women in China: an age-period-cohort analysis

**DOI:** 10.3389/fpubh.2025.1608189

**Published:** 2025-08-26

**Authors:** Hui Shen, Yongxiang Xie

**Affiliations:** ^1^Department of Sociology, Fudan University, Shanghai, China; ^2^School of Educational Science, Anhui Normal University, Wuhu, China

**Keywords:** domestic violence, women, rural, urban, age-period-cohort effect

## Abstract

**Objective:**

Domestic violence (DV) against women is a worldwide public health problem. This study explored the dynamics of DV in China from 1990 to 2010.

**Methods:**

Based on nationally representative data from the 1990, 2000, and 2010 China Women's Social Status Survey (CWSS) involving 29,995 women, we employed the Hierarchical APC-Cross-Classified Random Effects Models (HAPC-CCREM) to disentangle the effects of age, period and cohort on DV trends.

**Results:**

The reported overall prevalence of DV substantially declined from 26.7% in 1990 to 5.4% in 2010. The decline was more pronounced in rural areas (from 31.9 to 7.8%) than in urban areas (from 21.4 to 3.2%). The highest prevalence of reported violence occurred among women aged 30–34. However, among rural women, the risk increased with age. The period effect revealed a consistent decline in women's risk of DV over time, with rural areas showing a faster reduction than urban areas. The cohort effect indicated a significant decrease in risk for women born between 1976 and 1990 compared to earlier cohorts. Among urban women, the risk remained relatively stable across cohorts, whereas rural women experienced a marked decline.

**Conclusions:**

Overall, the risk of DV against women showed a downward trend. Distinct age, period, and cohort effects were observed, with a higher risk among women aged 30–34 and a lower risk among those born after 1975. The disparity in DV risk between urban and rural women narrowed over time and across birth cohorts. These patterns may be linked to broader shifts such as anti-domestic violence legislation, public health education, and improvements in women's socio-economic status.

## 1 Introduction

Intimate partner violence (IPV) is a “global pandemic” (1). The World Health Organization estimates that about 30% of women worldwide have experienced physical or sexual violence from a partner in their lifetime (2). The pervasive problem poses serious public health and social challenges, with the overwhelming burden falling on women. It severely undermines women's physical, mental, and reproductive health (3), threatens the stability of intimate relationships (4), and harms the health and well being of children (5). Whether IPV against women is declining remains an open and important question. While some high-income regions report declining rates of such violence (6), developing countries continue to face significant challenges, with evidence suggesting stagnant or increasing prevalence (7, 8). Although China is the world's most populous developing country and a major emerging economy, little is known about the trends in IPV within its borders (9, 10). In the Chinese context, “Domestic Violence” (家庭暴力) typically refers to intimate partner abuse, especially that occurring within marriage (10). China's rapid economic and social development has brought about profound changes in family structures and marital relationships (11). Therefore, analyzing the dynamics of domestic violence (DV) against women in China is essential for informing evidence-based prevention strategies in other developing countries.

Previous studies have analyzed the prevalence of violence against women in China using cross-sectional data, and two notable reviews have synthesized empirical findings across different time periods. Tang and Lai (12) analyzed 19 empirical studies conducted between 1987 and 2006, reporting average lifetime prevalence rates of 19.7% for overall abuse, 42.6% for psychological, 14.2% for physical, and 6.7% for sexual violence. A more recent review by Yang et al. (10), covering Chinese- and English-language publications from 1997 to 2016, summarized the lifetime prevalence in the general population ranged from 17.4 to 24.5% for psychological violence, 2.5%−5.5% for physical violence, and 0.3%−1.7% for sexual violence. Although the two reviews suggest a possible decline, wide variations in sampling and measurement across the underlying studies hinder meaningful comparisons. Longitudinal data are essential to assess whether the observed patterns reflect actual temporal trends in partner violence.

Moreover, trends in violence against women are shaped by the combined effects of age, period, and cohort factors. Age effects represent aging-related developmental changes within individuals; in this study, they specifically refer to shifts in marital relationships associated with aging. Period effects capture the influence of external social conditions that affect women's risk of partner violence during specific time periods. Cohort effects reflect shared experiences of birth cohorts that influence their vulnerability in distinct ways (13, 14). Previous research has not fully accounted for all these dimensions, making it difficult to accurately assess long-term trends (15). To address these limitations, this study draws on nationally representative longitudinal surveys from China (1990–2010) and applies Age-Period-Cohort models to examine three questions: how does the risk of partner violence change with age? How do historical periods shape these risks over time? Do women from different birth cohorts exhibit distinct patterns of victimization? Given substantial differences in socioeconomic development and gender equality between urban and rural China (16), this study then analyzes urban and rural samples separately.

## 2 Literature review

### 2.1 Age effects

Age is a key factor related to the risk of DV, and evidence suggests the violence often begins early in the course of a marital relationship. A study across 30 developing countries found that, among ever-married women who had experienced spousal abuse, the first incident occurred, on average, 3.5 years after the union formation (17). Similarly, data from the United States show that spousal violence is most prevalent among women in their mid-20s to early 30s (15). The elevated risk of DV around this stage may reflect the influence of the family life cycle, particularly the transition to parenthood. After childbirth, couples invest more time in childcare, leaving less for their relationship, which may reduce marital quality and increase conflict (18). Parenting introduces cumulative stressors into the household. First, raising young children often increases economic strain and heightens psychological distress for both spouses (19). This strain can further diminish marital satisfaction (20). Second, the transition from partner to parents typically escalates the burden of housework and childcare. Unequal distribution of responsibilities can trigger perceptions of unfairness, exacerbating marital tensions and conflict (21).

Beyond the early 30s, the relationship between age and women's risk of IPV remains inconclusive. Studies from the United States and Germany suggest that past-year IPV tends to decline with age (15, 22). However, in India, while earlier waves of National Family Health Survey showed a decreasing trend, the most recent data indicate the highest IPV rates among women aged 40–49 (23), suggesting a possible resurgence in midlife. In China, analyzing lifetime IPV by age requires considering urban–rural differences. Although IPV is more common early in marriage, women's economic autonomy and tolerance for violence vary by region, leading to divergent age patterns. In urban areas, more educated women are likely to seek help or leave violent marriages. For example, a 1990s Beijing study found that highly educated women were more inclined to seek support or divorce (24). This phenomenon of selective marital exit (i.e., the tendency of economically independent women to terminate violent marriages) may explain why fewer older women report DV. By contrast, rural women, often economically dependent on their husbands, may endure abuse to avoid losing housing or financial security (25, 26). This structural constraint leads to cumulative exposure, making older rural women more likely to report lifetime IPV. Accordingly, we propose the following hypothesis:

*H1: DV risk peaks at early 30s; after this point, it increases in rural areas but declines in urban areas*.

### 2.2 Period effects

Prior studies have reported divergent period trends in DV across different countries. In some countries, DV rates have risen over time due to incomplete legislation or poor policy enforcement (27, 28), while other countries have documented declines, often attributed to legal reforms, improved social services, and increased public awareness (29, 30). In the Chinese context, significant legislative progress has been made since the early 1990s. The 1992 Law on the Protection of Women's Rights and Interests marked the first legal recognition of DV as a social problem. The 2001 amendment to the Marriage Law officially listed DV as grounds for divorce, and in 2008, multiple government departments jointly issued guidelines on preventing and curbing domestic violence. These reforms have played a pivotal role in institutionalizing protections and fostering a policy climate of zero tolerance toward DV. A recent causal inference study in Brazil found a 22% decline in assault-related hospitalizations among women after a anti-DV law (31), underscoring the potential of legal reforms to reduce violence risk.

Concurrently, the development of information and communication technologies (ICT) may also have facilitated DV prevention. Since gaining full Internet access in 1994, China has experienced rapid ICT expansion; by 2010, the country had 457 million Internet users–44% of whom were women—offering new avenues to empower women and potentially lower their risk of violence. This digital expansion has not only broadened women's access to information and support services, but also enhanced their agency by enabling them to recognize abuse, seek help, and make informed decisions to protect themselves (32, 33).

Importantly, the effects of legal reforms and ICT expansion may be more pronounced in rural areas, where women have historically faced more limited access to legal protection (34), and where ICT coverage has lagged significantly (35). These institutional and technological shifts may, therefore have contributed to a steeper decline in DV prevalence in rural settings relative to urban areas (31). Thus, the following hypothesis can be claimed:

*H2: From 1990 to 2010, the overall prevalence of DV declined more rapidly in rural areas than in urban areas*.

### 2.3 Cohort effects

Cohort effects refer to systematic differences in the risk of DV among women born in different years or who shared similar life events (13). Prior research offers mixed findings. A cross-national study of 25 low- and middle-income countries found that younger cohorts reported higher odds of IPV, likely due to increased awareness and reporting rather than higher actual prevalence (8). In contrast, other studies have found that younger cohorts face lower IPV risk, potentially due to improved household bargaining power and the influence of feminist movements (15). In China, two major institutional shifts may underlie cohort-based differences in DV risk. First, educational expansion has substantially improved women's access to personal and structural resources. The 1986 Compulsory Education Law and the 1998 Education Revitalization Plan led to widespread access to education. Among women born between 1983 and 1985, the gender gap in higher education reversed (36). Evidence from Peru suggests that each additional year of schooling reduces women's likelihood of experiencing IPV in the past year by 2 percentage points, and lifetime IPV by 4 percentage points (37).

Second, the one-child policy, introduced in the late 1970s, fundamentally reshaped gender dynamics. As families could only have once child, many parents—especially in urban areas–invested more in daughters' education (38). As a result, these high-educated daughters not only had greater economic autonomy, but also had higher expectations for marital relationships and lower tolerance for violence (39).

While the cohort effects indicate an overall decline in DV risk, the pace and magnitude of this decline may vary by region. In rural areas, where gender inequality has historically been more severe and protective resources more limited, external reforms—such as educational expansion and family planning policies—may have had a greater influence, which could have contributed to a sharper decline in DV risk across cohorts. In contrast, older cohorts of urban women already had relatively low baseline levels of DV (40), which may have reduced the marginal effects of these reforms and help explain the more modest decline over time.

*H3: Compared to older cohorts, younger women report a lower risk of DV, and this cohort difference is more marked in rural areas*.

## 3 Data and methods

### 3.1 Data

This study utilized data from the China Women's Social Status Survey (CWSS), a nationally representative survey jointly conducted by the All-China Women's Federation and the National Bureau of Statistics. As the earliest large-scale probability survey on gender issues in China, the CWSS offers comprehensive data on gender equality, women's development, and household status in China. This paper utilized the 1990, 2000, and 2010 waves to construct a repeated cross-sectional dataset spanning two decades of social transformation. The analytic sample comprised 29,555 first-time married women aged 20–64, as this group is legally eligible for marriage and considered of working age in China–making them most relevant to DV. After data cleaning, the final sample included 10,017 respondents from 1990, 8,616 from 2000, and 10,922 from 2010. The sample was stratified by residential location, with 14,557 respondents from rural areas and 14,998 from urban areas. The CWSS datasets used in this study are publicly available, anonymized, and de-identified secondary data. Data access was granted by the CWSS research team.

### 3.2 Variables

The dependent variable was whether a woman had experienced DV, specifically physical violence perpetrated by her husband. Although the wording of the survey questions varied slightly across years, the underlying meaning remains consistent. In 1990, respondents were asked, “Has your spouse ever hit you during a conflict?” In 2000, the question was phrased as, “Has your spouse ever hit you?” In 2000, the question was revised to, “Has your spouse ever hit you?” In 2010, it was further modified to, “Have you ever been beaten by your spouse?” Responses were coded as 0 for “no” and 1 for “yes.”

This study focused on three key trend variables: age, period, and cohort. The age variable was categorized into nine groups at 5-year intervals for individuals aged 20–64. The period variable represents the survey years: 1990, 2000, and 2010. The cohort variable was determined by respondents' birth years, with women born between 1926 and 1990 grouped into 11 birth cohorts at 5-year intervals. Due to the small sample sizes, those born between 1926 and 1935 and those born between 1980 and 1990 were merged into single cohorts, respectively.

This paper included three sets of control variables. The first set included life-course variables, such as age at marriage and number of children. The second set covered the socio-economic status, including the woman's employment status and years of schooling. It also examined husband's relative resource status, assessed through educational assortative mating. The third set consisted of demographic variables, such as nationality and province. Table 1 presents descriptive statistics for all variables averaged across the three waves.

**Table 1:** Descriptive statistics of all variables (CWSS 1990–2010).

**Variables**	**Total sample (*N* = 29,555)**	**Rural sample (*N* = 14,557)**	**Urban sample (*N* = 14,998)**
	**Mean (SD) or %**	**Mean (SD) or %**	**Mean (SD) or %**
**Domestic violence**			
No	82.41	78.52	0.862
Yes	17.59	21.48	13.82
Average age	40.277 (0.062)	39.544 (0.088)	40.989 (0.086)
**Periods**			
1990	33.89	34.49	33.31
2000	29.15	29.73	28.59
2010	36.95	35.78	38.10
**Cohorts**			
1926–1936	3.02	2.25	3.77
1936–1940	3.83	2.87	4.75
1941–1945	4.24	3.91	4.57
1946–1950	9.15	9.12	9.17
1951–1955	14.64	15.13	14.18
1956–1960	14.01	14.02	14.00
1961–1965	17.82	18.74	16.94
1966–1970	14.49	15.68	13.34
1971–1975	9.52	8.92	10.11
1976–1980	5.09	4.72	5.45
1980–1990	4.17	4.64	5.72
Age at marriage	22.467 (2.896)	21.499 (2.583)	23.411 (2.872)
Number of children	1.905 (0.007)	2.219 (0.010)	1.601 (0.009)
**Employment status**			
Unemployed	22.79	10.51	34.71
Employed	77.21	89.49	65.29
Years of schooling	6.861 (0.024)	4.859 (0.030)	8.708 (0.030)
**Educational assortative mating**			
Homogamy	53.87	56.44	51.38
Hypergamy	34.01	34.00	34.02
Hypogamy	12.12	9.56	14.60
**Nationality**			
Minority	7.63	9.41	5.89
Han	92.37	90.59	94.11
**Province**			
Major metropolitan cities	7.56	5.35	9.69
Eastern	35.21	34.86	35.55
Central	32.23	33.02	31.47
Western	25.00	26.77	23.28

### 3.3 Age-period-cohort model

As previously mentioned, the variation in women's exposure to DV may be influenced by three interrelated temporal effects: age, period, and cohort. However, these variables are perfectly collinear (age = period – cohort), making it challenging to estimate their effects independently. To address this identification problem, this study employed Hierarchical Age-Period-Cohort Cross-Classified Random Effects Models (HAPC-CCREM) to disentangle these trends. In the HAPC-CCREM framework, age was treated as a level-1 individual-level variable, while period and cohort were specified as level-2 macro-level variables with crossed random effects (13). Taking the analysis of the total sample as an example, models are set up as follows:

Level 1 model:


(1) $$\begin{array}{c} \;LogitPr\left(DV_{ijk}=1\right)=\beta_{0jk}+\beta_{1}URBAN_{ijk}+\beta_{2}AGE_{ijk}\\ +\beta_{3}{AGE\text{\_}MARRIAGE}_{ijk}+\beta_{4}CHILDREN_{ijk}+\beta_{5}EMPLOYED_{ijk}\\ \;+\beta_{6}{YEARS\text{\_}SCHOOLING}_{ijk}+\beta_{7}ASSORTATIVE_{ijk}\\ \;+\beta_{8}HAN_{ijk}+\beta_{9}PROVINCE_{ijk} \end{array}$$

Level 2 model:


(2) $$\begin{array}{c} \beta_{0ijk}=\gamma_{0}+\mu_{0j}+\nu_{0k},\mu_{0j}\sim \text{N}\left(0,\tau_{\mu }\right),\nu_{0k}\sim \text{N}\left(0,\tau_{\nu }\right) \end{array}$$

Combined model:


(3) $$\begin{array}{c} \;LogitPr\left(DV_{ijk}=1\right)=\gamma_{0}+\beta_{1}URBAN_{ijk}+\beta_{2}AGE_{ijk}\\ +\beta_{3}{AGE\text{\_}MARRIAGE}_{ijk}+\beta_{4}CHILDREN_{ijk}+\beta_{5}EMPLOYED_{ijk}\\ \;+\beta_{6}{YEARS\text{\_}SCHOOLING}_{ijk}+\beta_{7}ASSORTATIVE_{ijk}\\ \;+\beta_{8}HAN_{ijk}+\beta_{9}PROVINCE_{ijk}+\mu_{0j}+\nu_{0k} \end{array}$$

for

*i* = 1, …, *n*_*jk*_ individuals with cohort *j* and period *k*;

*j* = 1, …, 13 birth cohorts;

*k* = 1, 2, 3 survey years.

In model (1), β_0*jk*_ represents the average log odds of all women in birth cohort *j* and survey year *k*. These odds vary by cohort and period, forming the level 2 model. In the full model (3), γ_0_ is the intercept, representing the average log odds of DV for all rural or urban women. μ_0*j*_ represents the cohort effect for cohort *j*, i.e., its average impact on β_0*jk*_ over time. It follows a normal distribution with a mean of 0 and variance τ_μ_. ν_0*k*_ represents the period effect for period *k*, i.e., its average effect on β_0*jk*_ over cohorts. This also follows a normal distribution with a mean of 0 and variance τ_ν_. Additionally, β_0*j*_ = γ_0_+μ_0*j*_ denotes the cohort effect for cohort *j* across periods, while β_0*k*_ = γ_0_+ν_0*k*_ represents the period effect for period *k* across cohorts.

All models were estimated using Stata 16. Model fit was evaluated using Deviance, the Akaike Information Criterion (AIC), and the Bayesian Information Criterion (BIC) (41).

## 4 Results

### 4.1 The overall trend in DV

**Figure 1:**
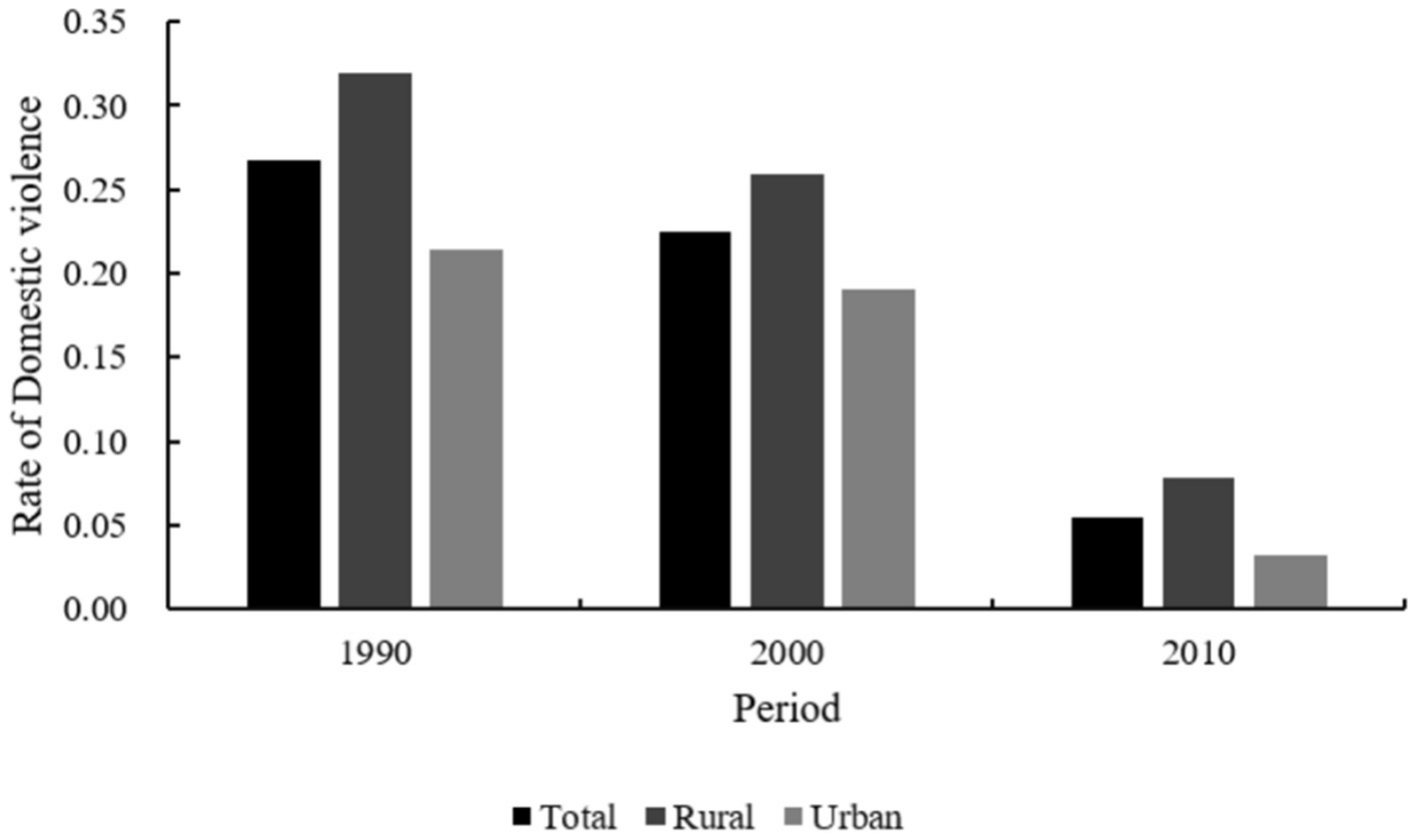
Overall trends in domestic violence against women, 1990–2010.

Figure 1 displays the trends in women's exposure to DV across the 1990, 2000, and 2010 survey waves. Overall, the risk of DV among Chinese women declined significantly from 26.7% in 1990 to 5.4% in 2010. The likelihood of rural women experiencing violence decreased more rapidly than that of urban women, with the risk falling from 31.9 to 7.8% for rural women, and from 21.4 to 3.2% for urban women.

Table 2 presents the estimated results for the total sample, as well as separate estimates for rural and urban subsamples. After controlling potential confounders, the results for the total sample (Column 1) show that urban women were less likely to experience DV than rural women. The likelihood of urban women suffering from DV was 14.3% (1-e^−0.154^) lower than that of rural women. The 95% CIs for the variance of the period and cohort effects in the model shown in Table 1 include 0, indicating that both the period and cohort effects were significant.

**Table 2:** HAPC- CCREM models of domestic violence.

**Variables**	**Total sample (*N* = 29,555)**	**Rural sample (*N* = 14,557)**	**Urban sample (*N* = 14,998)**
**Fixed effects**	**Coef. (SE)**	**Coef. (SE)**	**Coef. (SE)**
Urban	−0.154^***^ (0.041)	–	–
**Age (ref. 20–24)**			
25–29	0.262^†^ (0.159)	0.351^*^ (0.147)	0.334 (0.208)
30–34	0.430^***^ (0.101)	0.465^***^ (0.115)	0.410^*^ (0.168)
35–39	0.229 (0.171)	0.468^**^ (0.153)	0.138 (0.222)
40–44	0.211 (0.132)	0.324^*^ (0.137)	0.119 (0.190)
45–49	0.112 (0.192)	0.416^*^ (0.171)	−0.111 (0.230)
50–54	0.156 (0.171)	0.341^*^ (0.168)	−0.103 (0.217)
55–59	0.096 (0.214)	0.457^*^ (0.191)	−0.389 (0.239)
60–64	0.111 (0.209)	0.436^*^ (0.198)	−0.413 (0.251)
Age at marriage	−0.046^***^ (0.007)	−0.046^***^ (0.009)	−0.043^***^ (0.011)
Number of children	0.046^*^ (0.019)	0.036 (0.023)	0.049 (0.036)
Employed	0.054 (0.047)	0.135 (0.083)	0.001 (0.061)
Years of schooling	−0.070^***^ (0.005)	−0.052^***^ (0.007)	−0.092^***^ (0.009)
**Educational assortative mating (ref. homogamy)**			
Hypergamy	−0.185^***^ (0.036)	−0.113^*^ (0.047)	−0.256^***^ (0.057)
Hypogamy	0.318^***^ (0.055)	0.191^*^ (0.084)	0.403^***^ (0.074)
Han	0.296^***^ (0.065)	0.350^***^ (0.081)	0.160 (0.106)
**Province (ref. Major metropolitan cities)**			
Eastern region	0.254^**^ (0.082)	0.188^†^ (0.114)	0.310^*^ (0.121)
Central region	0.593^***^ (0.082)	0.535^***^ (0.115)	0.652^***^ (0.121)
West region	0.746^***^ (0.085)	0.679^***^ (0.120)	0.835^***^ (0.123)
Intercept	−1.279^*^ (0.500)	−1.529^**^ (0.509)	−1.076^†^ (0.594)
**Random effects variance** ^a^			
Period	0.583 (0.480)	0.531 (0.437)	0.859 (0.353)
Cohort	0.048 (0.029)	0.020 (0.015)	0.113 (0.093)
AIC	24,437.40	13,785.51	10,639.89
BIC	24,619.86	13,944.81	10,799.82

^†^*p* < 0.1; ^*^p < 0.05; ^**^p < 0.01; ^***^p < 0.001.

^a^The 95% CI for all variances of the period and cohort effects do not include 0.

**Figure 2:**
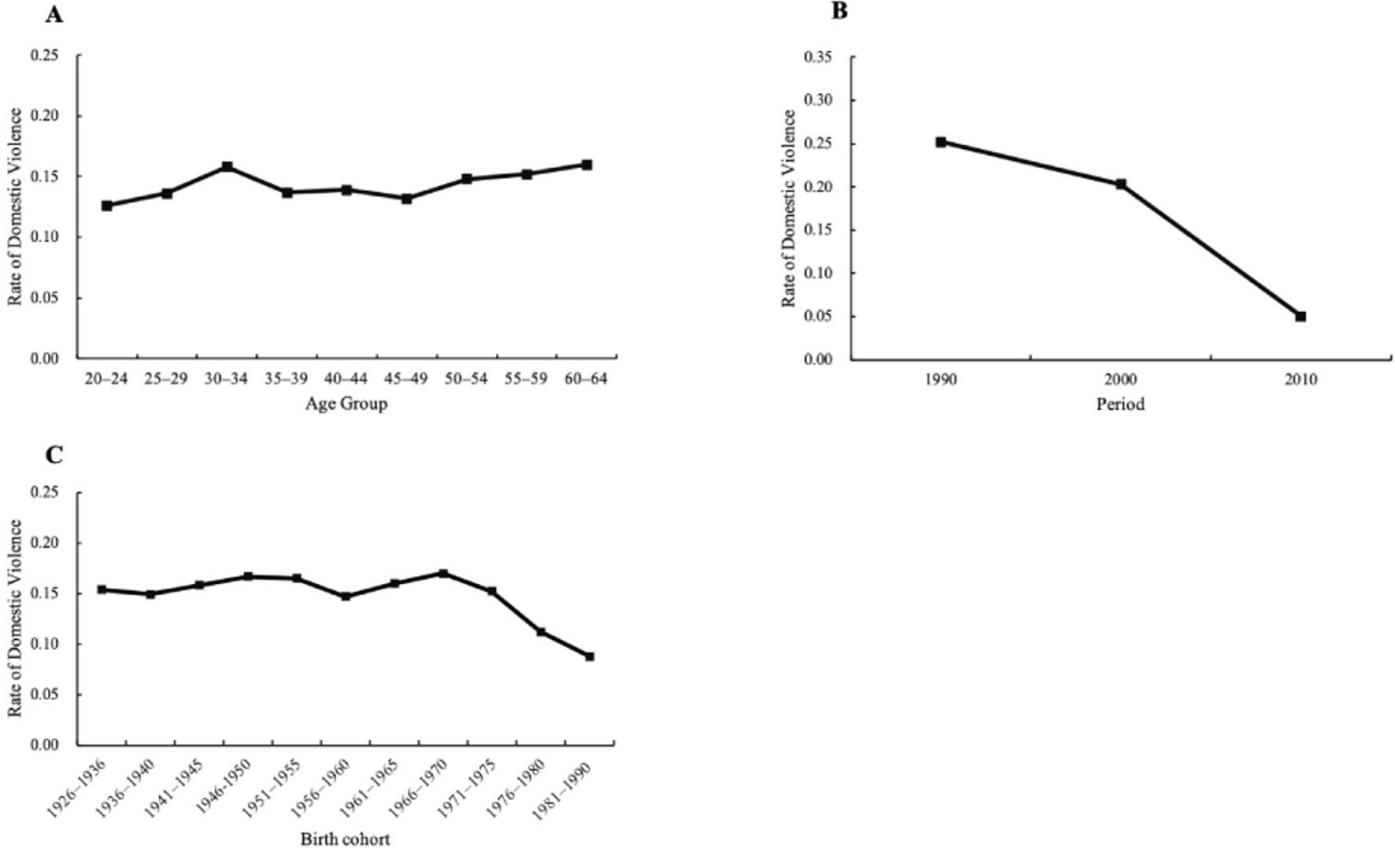
HAPC-CCREM analysis for all women, 1990–2010: **(A)** Age effects, **(B)** Period effects, **(C)** Cohort effect on domestic against women.

**Figure 3:**
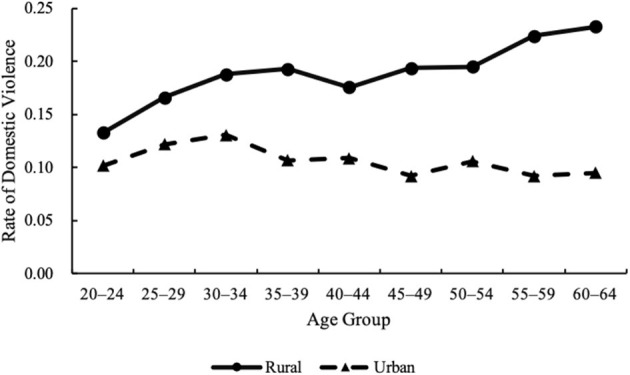
Age effects on domestic violence against rural and urban women, 1990–2010.

**Figure 4:**
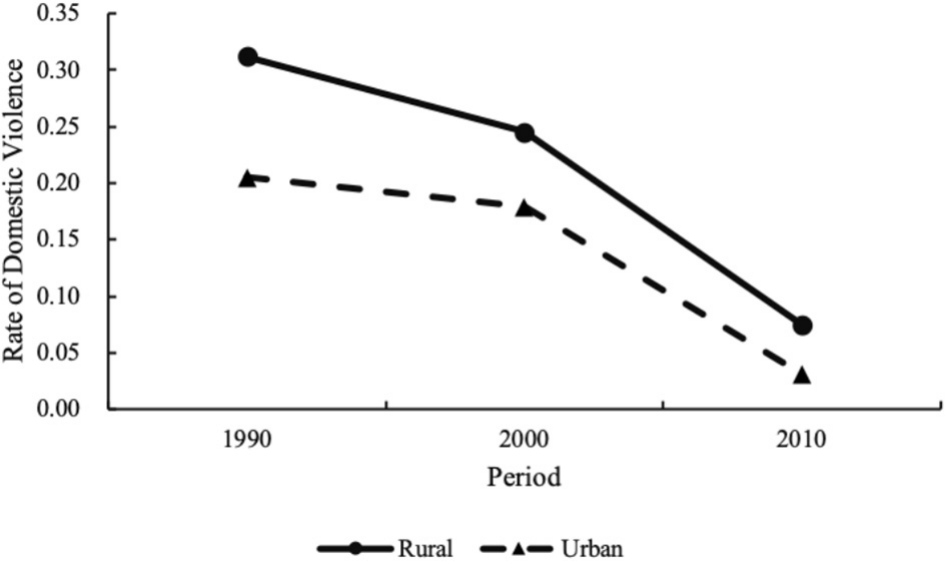
Period effects on domestic violence against rural and urban women, 1990–2010.

**Figure 5:**
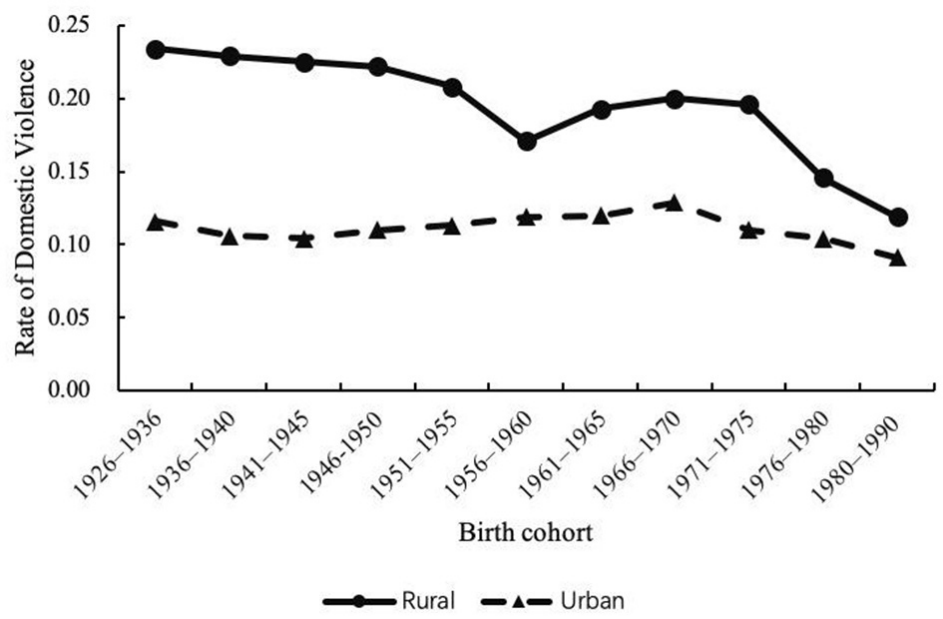
Cohort effects on domestic violence against rural and urban women, 1990–2010.

Figure 2 shows the estimated age, period, and cohort effects for the total sample of women. Figures 3–5 further disaggregate these patterns by urban and rural settings, revealing distinct trajectories (Table 2, Column 2–3). The following section integrates findings from both the full sample and the urban–rural subsamples to analyze these trends in greater detail.

### 4.2 HAPC-CCREM analysis of trend in DV

#### 4.2.1 Age effects

For H1, this study examined the age-related patterns of DV risk among women. As shown in Figure 2 (Panel A), the age trend for full sample can be divided into three stages: (1) a rapid growth stage (20–34 years); (2) a slow decline stage (35–49 years); and (3) a slow increase stage (50–64 years). However, based on model estimates (Table 2, Column 1), only the increase in DV risk up to age 34 was statistically significant. Therefore, women aged 30–34 exhibited the highest risk of experiencing DV, with the rate reaching 15.8%.

Further analysis in Figure 3 reveals divergent age trajectories by residence. For rural women, the likelihood of experiencing violence increased with age in two stages: it rose sharply between ages 20 and 39, then slowed between ages 40 and 64. In contrast, urban women experienced the highest DV risk at ages 30–34, after which the risk declined, though the decrease was statistically insignificant (*p* > 0.1). These results partially support H1.

#### 4.2.2 Period effects

For H2, regarding the period effect, Panel B of Figure 2 shows that the risk of DV among all women declined steadily from 1990 to 2010, based on model-estimated results.

Figure 4 further displays the period-specific trends in rural and urban areas. Over the two decades, both rural and urban areas experienced substantial reductions in DV incidence: in rural areas, the prevalence of DV dropped from 31.2% in 1990 to 7.5% in 2010, while in urban areas, it declined from 20.5 to 3.1% over the same period. Although urban women consistently reported lower DV rates, the rate of decline was more pronounced in rural areas. These findings fully support H2.

#### 4.2.3 Cohort effects

For H3, Figure 2 (Panel C) illustrates the overall cohort effect on DV for the full sample, while Figure 5 presents separate trends for rural and urban women. Overall, younger cohorts reported a lower risk of DV compared to older cohorts in both regions, although the decline patterns varied. In rural areas, DV risk dropped markedly from 23.4% for the oldest cohort (born 1926–1936) to 11.9% for the youngest cohort (born 1981–1990). The cohort effect curve exhibits three stages: (1) gradually declining cohort segment (1926–1960); (2) increasing cohort segment (1961–1975); and (3) rapidly declining cohort segment (1976–1990). In urban areas, cohort variation was less pronounced, with the highest risk at 12.9% for women born between 1966 and 1970 and a steady decline to 9.1% for the 1981–1990 cohort.

These findings support H3.

## 5 Discussion

### 5.1 Summary of major findings

#### 5.1.1 The overall trend in DV

This study examined the prevalence and trends of DV against women in China, drawing on nationally representative repeated cross-sectional survey data. Using data from the 1990, 2000, and 2010 CWSS, this study employed HAPC-CCREM models to assess the age, period, and cohort effects on women's DV experiences, along with differences between rural and urban areas. The findings help identify high-risk groups and may offer insights into how public health policies could potentially influence DV trends in China and other developing countries.

Our study found that from 1990 to 2010, the overall prevalence of DV against women in China declined markedly from 26.7 to 5.4%. This trend is consistent with findings by Fu et al. (42), who reported a decline in IPV-attributable deaths in China from 1990 to 2019. Unlike the increasing trends in developing countries such as Brazil (28), China's decline in women's exposure to IPV aligns more closely with patterns observed in most European countries and the United States (15, 30). This decline coincided with broader societal changes, including advances in women's rights legislation, expanded educational opportunities, and rapid economic development (17). Importantly, the urban-rural gap also narrowed during this period, with rural DV rates decreasing from 31.9 to 7.8% and urban rates from 21.4 to 3.2% between 1990 and 2010—findings consistent with earlier empirical studies conducted in China (10). Similarly, a Sweden study (43) found rural women to be more vulnerable to IPV. Taken together, these results raise the possibility that rural women may have been more exposed to or responsive to anti-domestic violence initiatives over the past decades.

#### 5.1.2 HAPC-CCREM analysis of trend in DV

Consistent with previous research, age remains a significant factor shaping women's risk of experiencing DV (17). This study identifies a general pattern in which women's risk of DV gradually increases between the ages of 20 and 34. The highest risk is observed among women aged 30–34, with a reported prevalence of 15.8%. This result aligns with studies in both China and the United States that highlight the vulnerability of women during this life stage (15, 42). Women aged 30–34 may be more vulnerable to violence due to physical and emotional exhaustion, financial stress from child-rearing, and conflicts over parenting approaches (42, 44). While the risk of violence against women in urban areas peaked between the ages of 30 and 34, it increased steadily with age in rural areas. This pattern may be shaped by differential levels of economic autonomy. Research in the United States showed financially dependent wives were more likely to remain with abusive husbands (45)–a pattern that also appears relevant in China. In rural areas, limited employment opportunities and restricted access to land or housing make women highly dependent on their husbands. Fear of losing basic living conditions may deter women from leaving abusive relationships, resulting in cumulative exposure over the life course. In contrast, urban women, especially those with higher education and independent income, are more likely to seek help or exit abusive marriages. The selective reduces the proportion of older women reporting DV in urban samples. Although market-oriented reforms and rural-to-urban migration have expanded access to non-agricultural employment for rural women, their participation rates and earnings remain lower than men's (46). This structural gender inequality continues to constrain autonomy and may sustain or exacerbate their vulnerability to DV.

In terms of the period effect, the risk of DV against women had steadily decreased over the 20 years. By 2010, the risk of DV was 19.8% of its level in 1990. This marked decline may be linked to broader institutional shifts, such as anti-domestic violence legislation, as supported by previous research (47). Since the 1990s, China has taken progressive steps to improve its legal framework addressing DV, aligning with broader international efforts, such as the 1992 Law on the Protection of Women's Rights and Interests and the 2001 Marriage Law amendment. The legislative reforms could increase women's awareness of their rights and improve their access to protection, potentially contributing to the observed downward trend in DV risk (15). Beyond legal reforms, the rapid development of ICT in China may be another factor associated with the decline in DV risk against women. Since gaining full Internet access in 1994, China has witnessed exponential growth in ICT coverage and usage; by 2010, the number of Internet users had reached 457 million. ICT may have contributed to disseminating DV-related public education, raising women's awareness of abusive behaviors, increasing legal literacy, and expanding access to online support platforms and community support (32, 33). Notably, the risk of violence against women declined more rapidly in rural areas than in urban areas, leading to a narrowing gap between the two. This pattern may be associated with the changing institutional and technological environments in rural regions, where legal reforms and ICT-based interventions–despite slower initial rollout–may have produced relatively pronounced effects by improving access to protection and information from a lower starting point (31).

Regarding the cohort effect, our data revealed a general decline in the risk of violence from the 1926–1936 to the 1981–1990 birth cohorts. However, this decline was not consistent. Among cohorts born between 1926 and 1975, the risk of violence fluctuated around 15%, followed by a sharp decrease for those born after 1975. This shift may coincide with China's reform and opening-up policy launched in 1978, which marked a major turning point in the country's socioeconomic landscape. This pattern contrasts with findings from Metheny and Stephenson (8), whose cross-national study of 25 low- and middle-income countries found that the youngest cohort of women faced the highest risk of experiencing IPV. In China, however, the youngest 1981–1990 cohort exhibited the lowest rate (8.8%). One possible explanation lies in expansive educational reforms initiated in 1986. A series of policies significantly improved women's access to education and even led to a reversal of the gender gap in education attainment (36). Education is widely shown to enhance women's resources and autonomy, thereby reducing their risk of DV (30). In addition, the one-child policy, introduced in the late 1970s and early 1980s, may have contributed to the decline in risk of DV across cohorts. With only one child permitted, families increasingly invested in daughters' education and well being (38). Women from these cohorts were more likely to attain higher education and economic independence, both of which are associated with lower tolerance for violence and higher expectations for egalitarian marital relationships (48). These changes may partially explain the lower DV risk among women born after 1980. Disaggregated by residency, our analysis exhibited a more substantial decline in DV rate among rural women (from 23.4 to 11.9%) compared to their urban counterparts (from 11.6 to 9.1%). This disparity suggests that rural women may have benefited more from recent social and policy changes, particularly as they started from a position of greater disadvantage. This cohort effect also aligns with period effects, where rural areas showed a steeper decline in DV risk–possibly reflecting stronger responsiveness to structural changes in disadvantaged contexts.

However, not all cohorts experienced steady improvement. Notably, women born between 1961 and 1975–particularly in rural areas–showed an uptick in DV risk. This group largely entered marriage during the 1980s and 1990s, a period of profound socioeconomic transformation in China driven by market-oriented reforms. In rural areas, the implementation of the Household Responsibility System boosted agricultural productivity but often rendered married women “landless.” In some regions, women received only 50%−70% of land allocated to men (49), significantly weakening their bargaining power within households and increasing their vulnerability to DV (25). Meanwhile, urban reforms, such as the restructuring of state-owned enterprises led to widespread unemployment. Whether women lost jobs and, thus economic autonomy, or their husbands faced unemployment and increased household financial strain, both scenarios may have heightened the risk of DV (50).

### 5.2 Practical implications

These findings carry important implications for DV prevention and intervention strategies in China and other developing countries. The marked decline in DV prevalence–particularly in rural areas–coincides with major public health efforts, such as the enactment of anti-domestic violence legislation, which may have contributed to this trend. However, persistent urban-rural disparities highlight the need for regionally tailored interventions. In this regard, interventions should prioritize expanding employment opportunities, strengthening social safety nets, and promote public education campaigns aimed at challenging gender norms, particularly in socioeconomically disadvantaged rural areas.

The elevated DV risk among women aged 30–34 underscores the necessity of age-targeted programs that alleviate financial and emotional stress associated with child-rearing and enhance access to family and community-based social support systems. The upward trend of DV risk with age among rural women calls for sustained and age-sensitive empowerment initiatives, particularly those addressing long-term economic vulnerability and social isolation. The steady decline in DV risk over period may reflect the cumulative effects of strengthened legal protections and expansion of public health education via ICT. Finally, the cohort-based findings suggest that the expansion of educational opportunities, along with the one-child policy, have contributed to a reduced DV risk among younger women–primarily by promoting greater parental investment in daughters' education and economic autonomy. Sustained investment in girl's education and gender equality initiatives remain essential to further reduce DV prevalence and promoting progress in women's health and well being.

### 5.3 Limitation

This study has several limitations. First, it relies on data ending in 2010, which limits its ability to reflect recent trends in domestic violence. While our analysis reveals a marked decline in DV prevalence from 1990 to 2010—likely reflecting early impact of legal reforms and gender equality efforts—these findings should be understood within a historical context. Since 2010, China has undergone major legal and social transformations, including the enactment of the Anti-Domestic Violence Law in 2016, which formally defining domestic violence and offering legal safeguards for victims. Preliminary evidence suggests this law has improved women's well being, such as increasing life satisfaction among married women (51). However, challenges remain. For instance, large-scale lockdowns during the COVID-19 pandemic may have increased risks by exacerbating household tensions and limiting access to external support (52). Besides the short-term impacts, the long-term consequences of the pandemic on gender-based violence remain an open question. It is yet to be determined whether women disproportionately affected by the pandemic were able to regain security after restrictions eased, or whether they continue to face heightened vulnerability.

This study's measurement of domestic violence is limited to physical abuse, excluding emotional and sexual forms. This narrow focus restricts the comprehensiveness of our analysis and may underestimate the true prevalence of DV.

Third, while the Age-Period-Cohort analysis reveals important temporal patterns in DV risk, these findings are descriptive and should not be interpreted as causal. Discussion of potential drivers–such as legal reforms, ICT development, educational expansion or one-child policy–are intended as contextual explanations rather than direct causal inferences. Moreover, DV is shaped by a complex interplay of structural and individual-level determinants, some of which may not have been fully accounted for in the current analysis.

## 6 Conclusion

This study employed HAPC-CCREM models to evaluate the age, period, and cohort effects underlying the temporal trends of DV risk against Chinese women during 1990–2010. Overall, both rural and urban women experienced a declining trend in DV risk over the two decades. The net age effect revealed that women aged 30–34 faced the highest risk of DV, while among rural women, the risk continued to rise with age. A significant period effect was observed, with DV risk decreasing over time, particularly in rural areas. In terms of cohort effects, women born after 1975 exhibited markedly lower DV risk and the risk of DV among rural women significantly decreased with those born in later years. These findings suggest the need for targeted interventions for women in their thirties and older rural women. To sustain and potentially reinforce the downward trend in DV, continued efforts to policies such as anti-domestic violence legislation, improving women's educational attainment, promoting gender equality, and expanding ICT infrastructure may be essential.

## Data Availability

The data used in this study are collected for restricted use. Anyone interested in the raw data can apply for access under approval of All-China Women's Federation and the National Bureau of Statistics. Further inquiries may be directed to the corresponding author.
